# Recovery of clinically relevant multidrug‐resistant *Klebsiella pneumoniae* lineages from wastewater in Kumasi Metropolis, Ghana

**DOI:** 10.1111/1758-2229.70018

**Published:** 2024-11-08

**Authors:** Amen Ekhosuehi, Odion O. Ikhimiukor, Helen Michelle Korkor Essandoh, Nana Yaw Asiedu, Isoken Tito Aighewi, Gabriel Temitope Sunmonu, Erkison Ewomazino Odih, Anderson O. Oaikhena, Dorothy Cyril‐Okoh, Clara Yeboah, Iruka N. Okeke

**Affiliations:** ^1^ Regional Water and Environmental Sanitation Centre, Kumasi Kwame Nkrumah University of Science and Technology Kumasi Ghana; ^2^ Department of Pharmaceutical Microbiology, Faculty of Pharmacy University of Ibadan Ibadan Nigeria; ^3^ Department of Biological Sciences University at Albany, State University of New York Albany New York USA; ^4^ Department of Civil Engineering Kwame Nkrumah University of Science and Technology Kumasi Ghana; ^5^ Department of Chemical Engineering Kwame Nkrumah University of Science and Technology Kumasi Ghana; ^6^ Department of Parasitology, Noguchi Memorial Institute for Medical Research University of Ghana Accra Ghana

## Abstract

Antimicrobial resistance (AMR) is under‐monitored in Africa, with few reports characterizing resistant bacteria from the environment. This study examined physicochemical parameters, chemical contaminants and antibiotic‐resistant bacteria in waste stabilization pond effluents, hospital wastewater and domestic wastewater from four sewerage sites in Kumasi. The bacteria isolates were sequenced. Three sites exceeded national guidelines for total suspended solids, biochemical oxygen demand, chemical oxygen demand and electrical conductivity. Although sulfamethoxazole levels were low, the antibiotic was detected at all sites. Multi‐drug‐resistant *Klebsiella pneumoniae* and *Pseudomonas aeruginosa were isolated* with multi‐locus sequence typing identifying *K. pneumoniae* strains as ST18 and ST147, and *P. aeruginosa* as ST235, all of clinical relevance. A comparison of ST147 genomes with isolates from human infections in Africa showed remarkable similarity and shared AMR profiles. Thirteen of the twenty‐one plasmids from ST147 harbored at least one AMR gene, including blaCTX‐M‐15 linked to copper‐resistance genes. Our study demonstrated high bacterial counts and organic matter in the analysed wastewater. The recovery of clinically significant isolates with multiple antibiotic and heavy metal resistance genes from the wastewater samples raises public health concerns.

## Graphical abstract

This study revealed a significant prevalence of antimicrobial resistance in *Klebsiella pneumoniae* and *Pseudomonas aeruginosa* isolates from wastewater, exhibiting diverse resistance profiles, including co‐resistance to antibiotics and heavy metals. These findings underscore the critical need for continuous One Health surveillance to mitigate the spread of AMR.

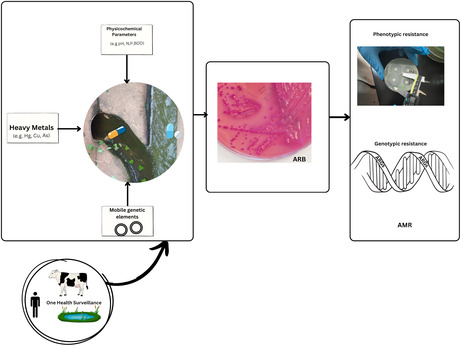

## INTRODUCTION

Addressing antimicrobial resistance (AMR) requires a comprehensive understanding of its scope, particularly in low‐income settings, where the burden is severe (Ikhimiukor et al., 2022; Okeke et al., 2024). The World Health Organization's (WHO) Global Action Plan has prompted many countries to enhance AMR surveillance and return data to the WHO Global Antimicrobial Resistance and Use Surveillance System (GLASS). In many African settings, where resources are limited, surveillance primarily targets human clinical isolates, focusing on WHO priority pathogens, including the ESKAPE‐E organisms: *Enterococcus feacium, Staphylococcus aureus*, *Klebsiella pneumoniae*, *Acinetobacter baumannii*, *Pseudomonas aeruginosa*, *Enterobacter* spp. and *Escherichia coli*. These pathogens are categorized by the urgency of new antibiotics development into critical, high and medium groups (Tacconelli et al., 2018). The surveillance often leverages on diagnostic microbiology at sentinel sites to identify these and other pathogens from sick patients.

To effectively combat the spread of priority pathogens, surveillance needs to extend beyond human clinical settings to include a holistic examination of antibiotic resistance genes and resistant isolates within a One Health context. Despite recognizing this need, AMR monitoring in low and middle‐income countries (LMICs) remains insufficient, typically skewed towards human and, to a lesser extent, animal health sectors (Ikhimiukor & Okeke, 2023; Munk et al., 2022; Okeke et al., 2024). In Ghana, for example, AMR monitoring is predominantly focused on human and animal samples, with limited research on environmental isolates (Osei Sekyere & Reta, 2020; Yevutsey et al., 2017). Furthermore, few studies or surveillance programs employ whole genome sequencing (WGS), which can provide detailed insights into the genetic basis of AMR and pathogen evolution (Vegyari et al., 2020). Therefore, characterizing the presence and distribution of AMR genes in environmental settings is increasingly crucial for effective AMR management.

Kumasi, Ghana's second largest city, grapples with significant challenges due to poorly maintained and dilapidated sanitation systems (UNICEF, 2016). Like many cities in LMICs, Kumasi's wastewater treatment infrastructure is inadequate, resulting in the discharge of untreated and poorly treated effluents into the environment. Wastewater represents a critical environment for the evolution of AMR, as it harbours a complex mix of pathogens, commensals, organic matter, heavy metals, nutrients, chemicals and antibiotic residues. While empirical evidence directly linking antibiotic residues pollution to proliferation of AMR genes in wastewater is scarce and challenging to obtain, the accumulation of these residues, coupled with favourable physicochemical conditions such as high nutrient levels and abundant microorganisms, likely create selective pressure that fosters the survival and proliferation of antibiotic resistant bacteria (Martínez, 2008; Osi et al., 2019). Additionally, the presence of heavy metals and disinfectants can also exert selective pressure on wastewater flora, complicating the assessment of antibiotic residues' specific impact on resistance genes development (Stanton et al., 2022; Tello et al., 2012). Some studies have demonstrated correlations between concentrations of antibiotic residues and the prevalence of antibiotic resistance genes. For instance, Kristiansson et al. (2011) discovered high levels of antibiotics, as well as elevated levels of resistomes and mobilomes, in river sediments receiving effluents from pharmaceutical industries. Similarly, Tello et al. (2012) used models to predict that concentrations of ciprofloxacin, erythromycin and tetracycline in river sediments and swine faeces could inhibit wild‐type bacterial populations by 60%–92%, thus favouring resistant bacteria that can colonize or infect humans. These data strongly suggest that wastewater can promote the selection of antibiotic resistant bacteria and genes, highlighting the need to track wastewater and other selection hotspots to support policy‐making (Booth et al., 2020; Fouz et al., 2020).

Priority pathogens that are opportunistic pathogens, such as *Klebsiella*, are well known sources and sinks of mobile resistance genes that can be transferred to other organisms (Wyres & Holt, 2018). This study assessed sulfamethoxazole (SMX) residues and investigated the presence of resistant bacteria and identified clinically relevant *K. pneumoniae* and *P. aeruginosa* species in raw wastewater and waste stabilization pond effluents in Kumasi, Ghana. Additionally, we measured selected physicochemical parameters of the raw wastewater and waste stabilization pond effluents.

## EXPERIMENTAL PROCEDURES

### Study site description

**FIGURE 1:**
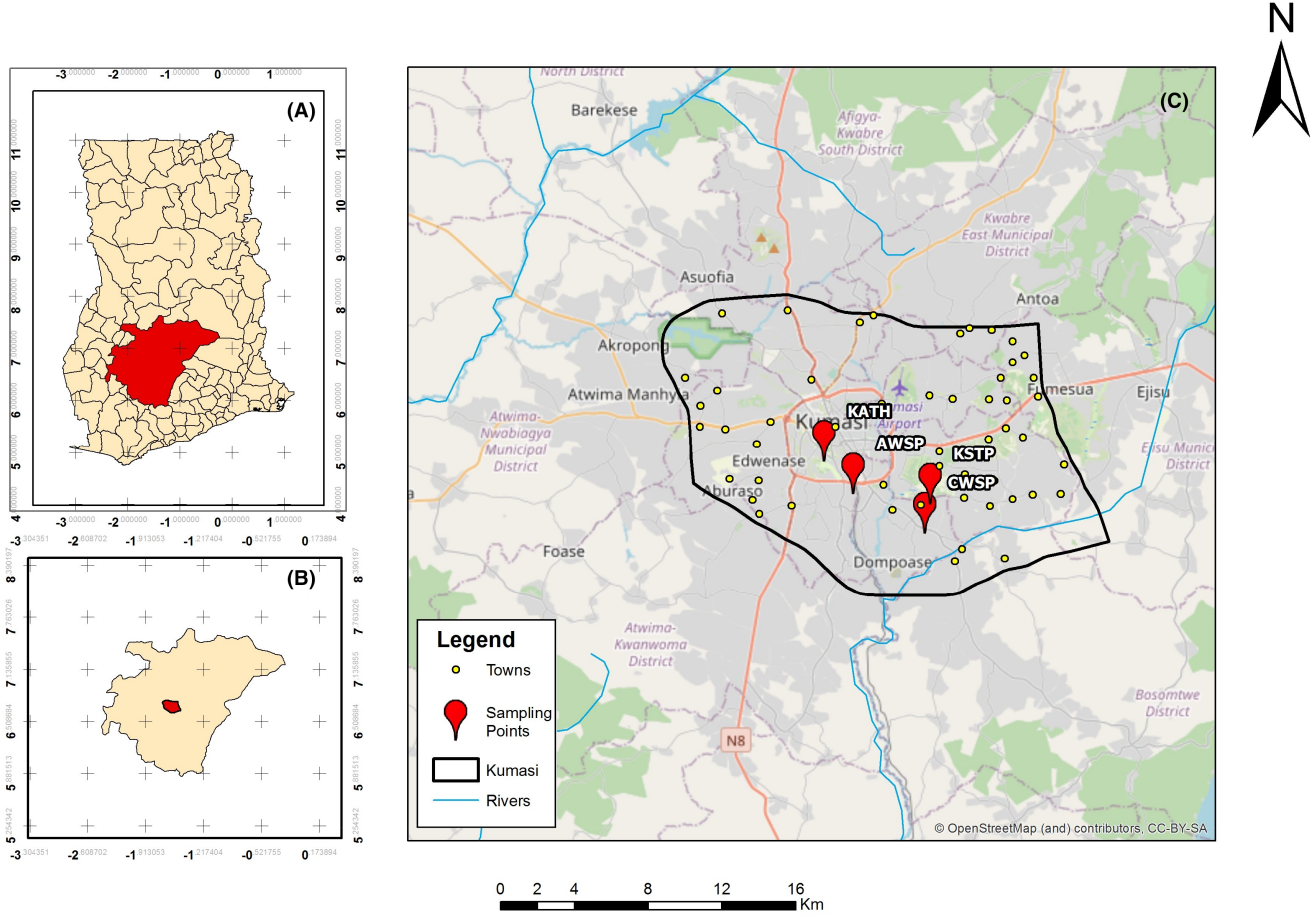
Study area (A) Ghana, (B) Ashanti region and (C) Kumasi metropolitan district.

In this study, treated wastewater samples were collected from Asafo (6.67336N, −1.61305E) and Chirapatre (6.654105N, −1.57824E) waste stabilization ponds (WSPs). Additionally, untreated wastewater samples were collected from the sewers of Komfo‐Anokye Teaching Hospital (KATH) (6.689105N, −1.62716E) and Kwame Nkrumah University of Science and Technology's sewage treatment plant (KSTP) (6.66833N, −1.5756E) before their discharge into a wetland (Figure 1). The selection of these sites was based on the presence of sewers infrastructure and accessibility.

Asafo WSP (AWSP) is situated in a densely populated, low‐income area in Kumasi. Originally designed in 1994 to serve 320 households with an estimated population of around 20,000, the AWSP initially comprised two anaerobic ponds, one facultative pond and two maturation ponds. In nearly 30 years of operation, the facility has experienced some minor breakdowns. Recent upgrades, including the addition of a second anaerobic pond to accommodate new connections from Kumasi Polytechnic hostels, reflect its ongoing adaptation (Salifu, 2013). AWSP discharges its treated effluent into the Subin river which flows through the city centre and joins the Oda river where it supports irrigation for local farming communities (Azanu et al., 2018). The Chirapatre WSP (CWSP) receives both sewage and household contaminants. Initially designed for 300 households with an estimated population of about 1800, CWSP has been expanded due to community growth (Darko et al., 2016; Tenkorang et al., 2012). It currently consists of an anaerobic pond, two facultative ponds and two maturation ponds. The latter of which are used for aquaculture. Effluents from CWSP flows into a nearby stream (Table 1).

**TABLE 1:** Site characteristics.

Sites	Wastewater contributors	Sampled effluent	Discharge point
AWSP	20,000 households	Treated	River
CWSP	300 households	Treated	Stream
KATH	KATH sewage	Raw	Natural wetland
KSTP	Student halls	Raw	Natural wetland

*Abbreviations*: AWSP, Asafo waste stabilization pond; CWSP, Chirapatre waste stabilization pond; KATH, sewage from Komfo Anokye Teaching Hospital Sewers; KSTP, sewage from Kwame Nkrumah University of Science and Technology's Sewage treatment plant.

KATH is the only teaching hospital in Kumasi with a capacity of 1200 beds (Azanu et al., 2018). It channels its sewage through a network of sewers that ultimately discharges into a natural wetland (see Table 1). Meanwhile, KSTP receives mainly sewage from eight halls of residence and annexes on the campus, serving over 7000 students (Awuah, 2014). Notably, during the sampling period, the trickling filter was undergoing maintenance, necessitating the rerouting of wastewater to a natural wetland.

### Sampling and sample collection

Flow‐proportional composite samples were manually collected for chemical and physicochemical analysis. To achieve this, three 6‐hourly aliquots were taken, each calculated based on flowrate at the time of sampling. These aliquots were then combined to create a 1 L sample for the analysis. The generated samples were kept on ice and transported to the laboratory within 12 h for processing. For microbiological analysis, 500 mL grab samples were collected in sterile autoclavable bottles. Duplicate samples were collected monthly from each sampling sites, resulting in a total of 120 samples collected between July and December 2019.

### Chemical and physicochemical analysis

SMX residues were analysed using solid phase extraction (SPE) followed by High Performance Liquid Chromatography coupled with a diode array detector (HPLC‐DAD) (Cecil Adept Binary Pump HPLC with WaveQuest DAD Detector). The analytical investigation adhered to the procedures outlined by Zhou and Jiang (2014) with some modifications. Wastewater samples (1 L) were first filtered using 240 mm Whatmann filter paper before undergoing cleanup via SPE. The hydrophilic–lipophilic balance (HLB) sorbents (Oasis HLB, 6 cc/500 mg, Waters) were preconditioned with 10 mL methanol and equilibrated with 15 mL distilled water. The prepared wastewater samples were then loaded onto the HLB sorbent fitted on a manifold connected to a vacuum pump. Elution of the sorbents was carried out using 10 mL methanol collected into 15 mL centrifuge tubes and dried under a nitrogen stream. The dried eluents were reconstituted with 2 mL methanol and vortexed at 3500 rpm for 10 min before analysis by HPLC‐DAD.

The mobile phase constituted 70% of acetonitrile (A) and 30% of 0.1% formic acid in distilled water (B). Column temperature was maintained at 35°C with a pump flow rate of 1 mL/min and detection was performed at a wavelength of 280 nm. Fifty microliters of the samples were injected into a reversed phase stationary phase column (Phenomenex, Synergi MAX‐RP 150 × 4.60, 4 μm). Additionally, samples were also analysed for pH and electrical conductivity (EC) on‐site using a multi‐parameter (Pc Testr 35, Eutech Instrument). Total suspended solids (TSS) were determined according to American Public Health Association's standards methods for examination of Wastewater (APHA, AWWA, WEF, 2017). Chemical oxygen demand (COD), total phosphorus (TP) and total nitrogen (TN) were determined using a spectrophotometer (HACH, DR 3900). Biochemical oxygen demand (BOD) was assessed using the manometric method (Velp scientifica BOD Evo Sensor and Lovibond BOD system).

### Chemical method validation

Chemical method validation was conducted in accordance with standard guidelines (Booth & Simon, 2016; International Council for Harmonisation, 2022). A 5‐point calibration curve for SMX (Sigma Aldrich) was constructed with concentrations ranging from 0.44 mg/L to 7.04 mg/L, yielding a regression coefficient of 0.99%. The limit of quantification (LOQ) and limit of detection (LOD) were calculated using a signal‐to‐noise ratio of 10 and 3 respectively. The calculated LOD and LOQ values for SMX were 0.02 mg/L and 0.1 mg/L respectively. Absolute recovery studies were also conducted using 1 L wastewater and 1 L distilled water in duplicates at the lowest and highest concentrations within the method calibration ranges. The absolute recovery was calculated as the ratio of the peak areas from wastewater to distilled water samples, were 68.75% and 60.97% respectively.

### Isolation and characterization of bacteria isolates

Total heterotrophic counts (THC) were determined according to American Public Health Association's standards methods for examination of Wastewater (APHA, AWWA, WEF, 2017). Samples were mixed to ensure uniform microbial dispersion, and serial dilutions ranging from 10^−2^ to 10^−6^ were prepared in sterile distilled water due to the high microbial load in sewage‐impacted wastewater. The diluted samples were then spread onto Plate Count Agar (PCA, Oxoid) in triplicate and incubated at 37°C for 24–48 h. Plates containing 25–300 colonies were used to compute the microbial counts. Single and separate colonies from each plate were subsequently sub‐cultured onto PCA and later into nutrient broth for cryopreservation. Isolates were subjected to Gram staining and a selected subset were further identified using the Gram‐negative (GN) test kit (Ref: 21341) on the VITEK 2 system (version 2.0, Marcy‐l'Etoile, France, Biomérieux). Isolates that had low‐confidence identities or were unidentified on VITEK2, as well as Gram positive strains, underwent 16S rRNA gene sequencing. DNA extraction was performed using MP Biomedicals FastDNA™ spin kit for soil following the manufacturer's protocol. Polymerase chain reaction (PCR) amplification of the 16S rRNA gene was conducted using 16S_1492r (5‐′GGTTACCTTGTTAGACTT‐3′) and 16S_27f (5′GAGAGTTTGATCCTGGCTCAG‐3′) primers designed by Turner et al. (1999) and Lane (1991) respectively. The PCR amplicons were visualized on a 1% agarose gel (CSL‐AG500, Cleaver Scientific Ltd.) stained with EZ‐vision® Bluelight DNA Dye and then Sanger‐sequenced using the Applied Biosystems ABI 3500XL Genetic Analyser. The sequencing outputs were converted from ABI chromatogram to FASTA format using the DNA Baser Assembler (https://www.dnabaser.com/download/download.html) and the sequences were assembled using the BioEdit Sequence Alignment Editor (https://bioedit.software.informer.com/7.2/). Bacteria identification was conducted by comparing the sequences to the 16S rRNA/ITS database nucleotide database of the National Center for Biotechnology Information (NCBI) using the BLASTN tool (https://blast.ncbi.nlm.nih.gov/Blast.cgi). Isolates from genera commonly associated with human infections were subjected to antimicrobial susceptibility testing and WGS.

### Antimicrobial susceptibility testing

Antimicrobial susceptibility testing was performed on eight clinically relevant isolates, comprising *K. pneumoniae* (*n* = 7) and *P. aeruginosa* (*n* = 1), using Biomérieux VITEK AST N280 cards (Ref: 413432) following the manufacturer's instructions. The VITEK AST N280 cards include a panel of 85 antimicrobials, such as imipenem‐relebactam, meropenem‐vaborbactam and an extended‐spectrum beta‐lactamase (ESBL) test. As the aim was to determine clinical significance of resistances, minimum inhibitory concentrations obtained for each antibiotic were interpreted using the breakpoints established by the Clinical and Laboratory Standards Institute (CLSI, 2021).

### Whole genome sequencing

DNA from *K. pneumoniae* (*n* = 7) and *P. aeruginosa* (*n* = 1) identified as clinically significant priority pathogens via VITEK2, was extracted using the Wizard DNA extraction kit (Promega; Wisconsin, USA) following the manufacturer's instructions. Quantification of the extracted DNA was performed on a Qubit fluorometer (Invitrogen; California, USA) utilizing the dsDNA broad range assay. Double‐stranded DNA libraries were prepared using NEBNext Ultra II FS DNA library prep kit for Illumina incorporating 96 unique indexes (New England Biolabs, Massachusetts, USA; Cat. No: E6609L). Subsequent quantification of the DNA libraries employed the dsDNA High Sensitivity assay on a Qubit fluorometer, and average fragment length of the DNA libraries were determined using 2100 Bioanalyzer (Agilent).

The libraries were sequenced using 150 bp paired‐end chemistry on an Illumina MiSeq (Illumina, California, USA). Sequence reads were assembled using the GHRU assembly pipeline (https://gitlab.com/cgps/ghru/pipelines/dsl2/pipelines/assembly) implemented in a Nextflow workflow. Briefly, the reads were trimmed using trimmomatric v0.39 (Bolger et al., 2014), and Cutadapt v3.2 (https://github.com/marcelm/cutadapt) was used to remove adapter sequences from the reads. Trimmed reads were de novo assembled with SPAdes v3.15.3 (Bankevich et al., 2012) and the quality of the assembled genomes was evaluated using QUAST v5.0.2 (Gurevich et al., 2013), ConFindr v0.7.2 (Low et al., 2019), qualifyr v1.4.4 (https://gitlab.com/cgps/qualifyr). Genomes with contamination levels <5%, number of contigs <300 and N50 > 25,000 were subjected to downstream analyses. Strain identities were confirmed using Bactinspector v0.1.3 (https://gitlab.com/antunderwood/bactinspector).

### Klebsiella *typing and determination of genetic determinants for AMR and virulence*

We used Kleborate v2.2.0 (Lam et al., 2021) to confirm *Klebsiella* species identities and determine multi‐locus sequence types. Determination of *Klebsiella* K‐ and O‐locus types were done using Kaptive v2.0.0 (Lam et al., 2021). Genotypic mechanisms of AMR were determined by screening the genome assemblies for the presence of AMR genes using AMRFinderPlus v.3.10.23 (Feldgarden et al., 2021) and its accompanying NCBI AMR database. The presence of genetic determinants for virulence in the genomes was detected by following GHRU protocols implemented on a nextflow workflow (https://gitlab.com/cgps/ghru/pipelines).

### Plasmid replicons and plasmid sequence reconstruction

Plasmid replicons in the genomes were detected using PlasmidFinder implemented in a Nextflow workflow (https://gitlab.com/cgps/ghru/pipelines). To investigate the potential presence of AMR genes on plasmids, we used the mob‐recon tool available in the MOB‐suites software to reconstruct plasmid sequences from the draft genome assemblies (Robertson & Nash, 2018). The plasmid contigs generated were used as input to determine presence of AMR genes and heavy metal resistance genes using AMRFinderPlus v3.10.23 (Feldgarden et al., 2021).

### Phylogenetic analysis of genomes

To elucidate the evolutionary relationships between our strains and others from same clonal lineage, we downloaded all publicly available *K. pneumoniae* ST147 genomes (*n* = 29) from Pathogenwatch (https://pathogen.watch/), including isolates from human (*n* = 28) and environmental (*n* = 1) sources across Africa (Algeria = 2, Egypt = 5, Kenya = 4, Nigeria = 13, South Africa = 1 and Zambia = 4) (Argimón et al., 2021). For *K. pneumoniae* ST18, we included all available genomes from Pathogenwatch in our phylogeny analysis. The genomes were first annotated using Prokka v1.14.6 (Seemann, 2014) and then subjected to pangenomes analysis using Panaroo v1.2.7 (Tonkin‐Hill et al., 2020). Multiple sequence alignment was performed with MAFTT v7.4.472 (Katoh & Standley, 2013) to generate a core genome alignment. Single nucleotide polymorphisms (SNPs) were extracted from this alignment using snp‐sites v2.5.1 (Page et al., 2016) and subsequently used in RAXML v8.2.12 (Stamatakis, 2014) to construct a phylogenetic tree employing GTR nucleotide substitution model and GAMMA distribution of heterogeneity. Pairwise SNP distances were calculated using snp‐dists v0.8.2 (https://github.com/tseemann/snp-dists), and the phylogeny was visualized on iTOL (Letunic & Bork, 2019).

### Statistical analysis

All data were entered in an Excel Spreadsheet and analysed using RStudio 2022.02.1. To compare the number of genetic determinants such as plasmid replicons, AMR and virulence genes identified in the *K. pneumoniae* ST147 strains from our study with those detected in human associated *K. pneumoniae* ST147 strains across Africa, we applied the Wilcoxon Rank Sum test. Differences in selected physicochemical parameters across various locations were evaluated using One Way Analysis of Variance (ANOVA). Where applicable, post‐hoc tests were conducted, with significance determined at *p* < 0.05 using SPSS software (version 25).

## RESULTS

### Wastewater pollution levels in sampling sites

The mean physicochemical parameters for all sampled sites are presented in Table S1. AWSP, KSTP and KATH demonstrated comparable physicochemical profiles, with no significant differences in pH, BOD/COD ratio, BOD, COD, EC, TN and TSS (*p* ≤ 0.05). The parameter ranges across the sites were as follows: pH, 7.77 ± 0.11–8.30 ± 0.24; BOD/COD ratio, 0.51 ± 0.12–0.60 ± 0.03; BOD, 96.17 ± 18.08–506 ± 39.99; COD, 162.41 ± 31.71–883.17 ± 71.28; EC, 1437.5 μS/cm ± 196.62–2074.83 μS/cm ± 241.53; TN, 73.93 mg/L ± 26.14–139.10 mg/L ± 31.06 and TSS, 70.17 mg/L ± 20.62–278 mg/L ± 44.25. When compared to the Ghana Environmental Protection Agency's (GEPA) limits, BOD, COD, EC, TN and TSS concentrations at AWSP, KSTP and KATH exceeded the respective thresholds of 50 mg/L, 50 mg/L, 1500 μS/cm, 50 mg/L and 50 mg/L. Notably, KSTP recorded the highest TP content of 8.05 mg/L ± 1.21 while TP levels at other sites were relatively the same. In contrast, CWSP displayed significantly lower values for most examined parameters, including BOD and COD.

SMX was consistently detected across all sites throughout the 6 months sampling period, albeit most concentrations were <LOQ. SMX levels ranged from <LOD to 0.127 mg/L, with the highest quantifiable value of 0.127 mg/L observed at KATH.

The mean THC for each sites are detailed in Table S1, ranging from 7.78 × 10^5^ cfu/mL to 2.50 × 10^7^ cfu/mL. The highest count was recorded at KSTP (2.50 × 10^7^ cfu/mL), while the lowest count was observed at CWSP (7.78 × 10^5^ cfu/mL). However, THC Counts from WSP effluents (AWSP and CWSP) did not significantly differ (*p* ≤ 0.05) from those in the untreated wastewater samples from KSTP and KATH. Only 21 isolates were identified due to limited resources and these were selected based on differences in colony morphology.

### Identification of wastewater isolates and whole‐genome sequencing of species of clinical significance

A total of 18 isolates were successfully identified biochemically using the VITEK2 system with three isolates remaining unidentified. The VITEK2 system also confirmed the identity of *Pseudomonas alcaligenes* (*n* = 1) from CWSP. Eight Gram negative bacteria were further confirmed to species level using WGS. Specifically, the identities of *K. pneumoniae* (*n* = 4) and *P. aeruginosa* (*n* = 1) from KATH and *K. pneumoniae* (*n* = 3) from CWSP (*n* = 1), KSTP (*n* = 1) and AWSP (*n* = 1) were confirmed.

In addition, 16S rRNA sequencing was employed to confirm the identities of nine isolates including *Bacillus* spp. (*B. stercoris* [*n* = 1] from KNUST; *Bacilus subtilis* [*n* = 2] from CWSP and AWSP respectively, and *B. velenzensis* [*n* = 2] from CWSP), *Enterococcus* spp. (*E. faecium* [*n* = 2] and *E. durans* [*n* = 1]) from AWSP, and *Exiguobaterium acetylicum* [*n* = 1] from CWSP. However, clinically relevant gram negative isolates, *K. pneumoniae* and *P. aeruginosa* were selected and analysed further.

The genomes of the seven *K. pneumoniae* isolates were of high quality, with contigs numbers ranging from 18 to 190 (average = 102), G + C contents between 57.09% and 57.56% (average = 57.29%), and N50 values from 86,833 bp to 1,266,334 bp (average = 314,159 bp) (Table S3). The *P. aeruginosa* genome, obtained from KATH, was also of high quality, assembling into 83 contigs with an N50 of 234,998 bp.

### Phenotypic and genotypic characterization of AMR

Amongst the seven *K. pneumoniae* isolates analysed, three were pan‐sensitive to the antimicrobials tested, while the other four exhibited resistance to fluoroquinolones, extended spectrum beta‐lactams and trimethoprim‐sulfamethoxazole, but were sensitive to imipenem and doripenem. Multilocus sequencing typing based on the allelic configurations of their seven housekeeping genes (*gapA*, *infB*, *mdh*, *pgi*, *phoE*, *rpoB*, *tonB*), identified the predominantly susceptible *K. pneumoniae* isolates as ST18 (*n* = 3; two from KATH, and one from CWSP). In contrast, the multidrug‐resistant *K. pneumoniae* were identified as ST147 (*n* = 4; two from KATH, one from AWSP and one from KSTP). Isolates within each sequence type demonstrated similar antibiotic susceptibility profiles (MIC cutoff values are presented in Table S2). ST18 isolates exhibited intrinsic resistance to ampicillin, as well as resistance to nitrofurantoin, whereas ST147 isolates were resistant to antimicrobials in seven drug classes including aminoglycosides, beta‐lactams, cephalosporins, fluoroquinolones, folate inhibitors, nitrofurantoin, quinolones, but were susceptible to carbapenems and tigecycline (Table S2).

ST147 isolates possessed a broad array of AMR genes. These included genes encoding resistance to aminoglycosides [*aadA1, aac(3)‐Iie, aph(3″)‐Ib, aph(6)‐Id*], aminoglycoside/quinolone [*aac(6′)‐Ib‐cr5*], beta‐lactam (*bla*_SHV‐11_, *bla*_CTX‐M‐15_, *bla*_OXA‐1_), biocides (*qacE*), fosfomycin (*fosA*), macrolides [*mphA, erm(B)*], phenicols (*catA1, catB3*), nitrofurantoin/quinolone exporter (*oqxA/oqxB*), quinolone (*qnrB2*), sulfonamides (*sul1, sul2*), in addition to multidrug efflux genes (*emrD*). The identified resistance genes corresponded to the observed phenotypic resistance patterns in the isolates (Table S3). In contrast, *K. pneumoniae* ST18 isolates harboured intrinsic and chromosomal AMR genes *bla*_SHV‐11_, *fosA*, *oqxA*, alongside *emrD*.

The *P. aeruginosa* isolate retrieved from the KATH sewer discharge pipe was resistant to ampicillin, ampicillin/clavulanic acid, extended‐spectrum beta lactams, fluoroquinolones, gentamicin and tigecycline. However, it was sensitive to amikacin, colistin and carbapenems. This extensively drug‐resistant *P. aeruginosa* isolate was classified as ST235 and carried AMR genes for aminoglycosides [*aph(3′)‐IIb, aac(6′)‐Ib, aadA1, aac(3)‐IIe*], beta‐lactams [*bla*_*OXA‐488*_, *bla*_*OXA*_,*bla*_*SCO‐1*_, *bla*_*TEM‐1*_, *bla*_*PDC‐35*_], phenicols [*catB7*, *floR2*], fosfomycin [*fosA*], antifolates [*sul1*, *dfrA14*] and tigecycline [*toprJ1*, *tmexD2*, *tmexC*]. Additionally, this strain exhibited resistance‐conferring mutations in the quinolone resistance‐determining regions of *gyrA* [T83I] and *parC* [S87L], as well as the plasmid‐mediated quinolone resistance gene *qnrVC1*.

### K. pneumoniae *isolates from wastewater in Kumasi are closely related to isolates from human infection elsewhere*

Two distinct capsular K‐locus (KL) configurations were detected in the *K. pneumoniae* genomes: KL10 was associated with ST147, and KL23 was identified in the genomes of ST18 strains. The O‐locus lipopolysaccharides types identified were O3/O3a in ST147 and O1/O2v2 in ST18 strains. A total of 58 virulence genes were identified using VirulenceFinder, most of which are associated with human virulence (Table S3). Of these, 43 virulence genes were conserved in both ST147 and ST18 genomes. Notably, a higher number of virulence determinants were observed in the ST18 genomes, ranging from 55 to 58, compared to 48 to 51 in the ST147 genomes. This variation in virulence gene content is attributed to the presence of a gene cluster exclusive to the ST18 genomes, which includes the *rfbABD* cluster (encoding O‐antigen biosynthesis enzymes), glycosyltransferase gene producing genes (KP1_RS17220, KP1_RS17225, KP1_RS17230) and DUF4422 domain‐containing protein (KP1_RS17240) (http://www.mgc.ac.cn/cgi‐bin/VFs/vfs.cgi?VFID=VF0561) (Table S3). Additionally, the enterochelin synthetase component D, (*entD*) was absent in the *entABCDEFS* operon in ST147. Importantly, none of the *K. pneumoniae* genomes from this study harboured key hypervirulence related genes (Kochan et al., 2022; Wyres et al., 2019).

**FIGURE 2:**
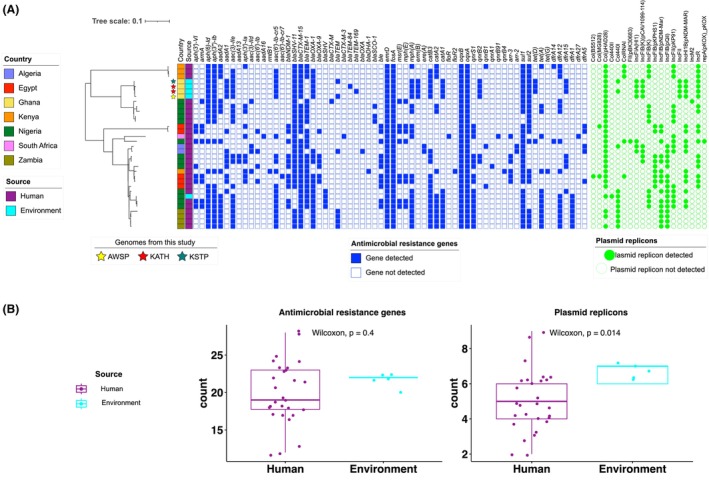
Genomic inferences from ST147 strains showing (A) phylogeny and gene presence/absence matrix of AMR genes and plasmid replicons in ST147 genomes from this study (Environment) and from genomes from other parts of Africa. (B) Boxplots showing relationship of AMR determinants and plasmid replicons in genomes from the AWSP—Asafo/waste stabilization pond, KATH—Adum/hospital sewer's discharge pipe, KSTP – KNUST/trickling filter. (This study) and from other parts of Africa.

Phylogenetic analysis revealed that the ST147 genomes from this study clustered closely with five genomes from Nigeria, isolated between 2013 and 2017 (Figure 2; Table S4). All ST147 genomes from this study were differentiated by ≤94 core genome pairwise SNPs (Table S5). A comparative analysis of AMR genetic determinants between our study and publicly available genomes from Africa (Afolayan et al., 2021; Carlos et al., 2021) (Figure 2), indicated that the majority of ST147 genomes (*n* = 31/33) carried the *bla*_CTX‐M‐15_ gene. However, only the genomes from our study and two genomes from Nigeria harboured the *qnrB2* gene. The number of AMR genes in *K. pneumoniae* ST147 from our study was not significantly different from those in clinical isolates from other African states (*p* = 0.4, 95% significance level) (Figure 2B).

**FIGURE 3:**
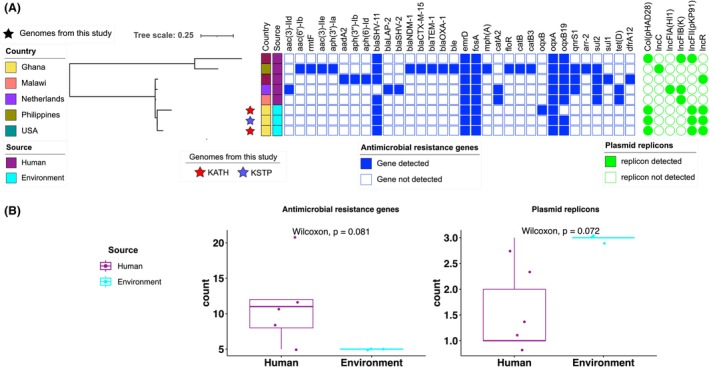
Genomic inferences from ST18 strains showing (A) Phylogeny and gene presence/absence matrix of AMR genes, plasmid replicons and number of virulence genes in ST18 genomes from KATH—Adum/hospital sewer's discharge pipe, KSTP—KNUST/trickling filter in this study and from genomes from Philippines and USA.

The only other *K. pneumoniae* ST18 genome identified in Pathogenwatch from Africa originated from Malawi. Our phylogenetic analysis included all available ST18 genomes, which also included isolates from the United States of America (*n* = 2), Netherlands (*n* = 1) and Philippines (*n* = 1) (Figure 3). There was no significant difference in the genetic composition of ST18 genomes from our study compared to those from human associated sources (*p* = 0.081, 95% significance) (Figure 3B). All ST18 genomes from this study were differentiated by ≤737 core genome pairwise SNPs.

### Plasmid replicons and plasmid reconstruction

A comprehensive analysis using PlasmidFinder identified eight distinct plasmid replicon types in the genomes from our study (Table S3). A comparison of number of plasmid replicon types between *K. pneumoniae* ST147 isolates from our study, bearing IncR, Col(pHAD28), IncFIA(HI1), IncFIB(K)(pCAV1099‐114), IncFII, IncHI1B(pNDM‐MAR) and Col440I (present in 2 genomes) replicons, and those associated with human infections in Africa revealed a significant difference (*p* = 0.014) (Figure 2B). In contrast, there was no significant difference in number of plasmid replicons from ST18 genomes from our study bearing IncFII(K) and IncR plasmid replicons and those from human infections (*p* = 0.072) (Figure 3B).

**FIGURE 4:**
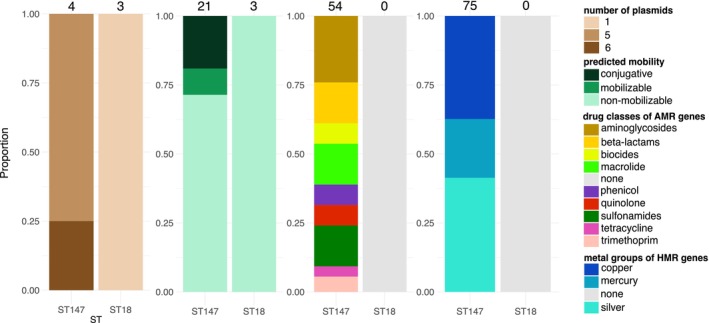
Details of plasmids sequenced based on MOB‐suites plasmid reconstruction tool, showing (A) proportion of number of plasmids reconstructed per genome. (B) Predicted mobility of reconstructed plasmids, (C) proportion of AMR (AMR) genes detected in reconstructed plasmids, (D) proportion of heavy metal resistance genes (HMR) encoding resistance to heavy metals detected in plasmids.

Using the mob‐recon tool, 24 putative plasmid sequences were reconstructed from the *K. pneumoniae* genomes in this study, including twenty‐one from ST147 and three from ST18. The reconstructed plasmids varied in number, with one plasmid present in each of the three ST18 genomes, five plasmids present in three ST147 genomes each, and six plasmids identified in a single ST147 genome. These plasmids were categorized into 11 distinct MOB‐suites primary plasmid clusters (Table S6). The plasmid predicted in ST18 was a 3.5 kb ColRNA replicon type belonging to MOB‐suites primary cluster AA116, and it did not harbour any antimicrobial or metal resistance genes. In contrast, the plasmid sequences in ST147 genomes, ranged from 3.4 to 115 kb in size. Thirteen of the twenty‐one ST147 plasmids contained at least one AMR gene specifying resistance to nine different classes of antimicrobial drug (Figure 4).

Further investigation revealed co‐resistance to antimicrobials and heavy metals in five of the reconstructed plasmids sequences (Table S6). Specifically, four plasmid sequences carried the complete copper resistance operon, *pcoABCDERS* together with AMR genes, including *bla*_CTX‐M‐15_, which was found in two of these plasmid sequences. Additionally, resistance to silver and mercury, were observed through the presence of the *sil* and *mer* gene operons, in five and four plasmid sequences, respectively.

## DISCUSSION

Wastewater surveillance is critical to understanding the effectiveness of decontamination and the risk that wastewater poses to proximal human populations. Physicochemical analysis of the examined sites revealed that the AWSP, KSTP and KATH sites were more polluted than CWSP. Although pH values at all sites met the GEPA limits, the levels of BOD, COD, EC, TSS and TN all exceeded the GEPA's stipulated guidelines for safe discharge into the environment (Owusu‐Ansah et al., 2015). Surprisingly, the physicochemical characteristics of AWSP did not significantly (*p* ≤ 0.05) differ from the untreated KSTP sample. Since WSP are designed to remove organic matter and pathogenic microorganisms (Lucas et al., 2021), the high BOD (222 mg/L ± 41.99) and COD (439.33 ± 76.72) values observed at AWSP after treatment, compared to GEPA limits suggests that AWSP may not be functioning optimally. Furthermore, the high mean TSS (133.33 mg/L ± 11.02) value obtained for AWSP samples, compared to GEPA's Standard of 50 mg/L for safe discharge of domestic wastewater effluents, and the isolation of multidrug‐resistant ST147 *K. pneumoniae* genetically similar to KATH isolates further point to a likely deficiency in the treatment plant. TSS, which measures the suspended solids, together with BOD is usually used to assess the effectiveness of treatment facilities (Vera et al., 2013). The BOD/COD ratio, which fell between 0.4 and 0.6 for all examined sites, indicated a high organic matter concentration in the wastewater. Biological wastewater treatment is normally associated with high bacterial counts (Modin et al., 2022), and the additional organic matter present in AWSP and CWSP samples would typically promote proliferation of microorganisms in wastewater. High organic matter, nutrients and pathogens are characteristic of raw sewage. Suboptimal wastewater treatment poses significant risks to public health, as it may serve as a breeding ground for the evolution and dissemination of antibiotic resistant bacteria and genes, and promote disease outbreaks (Mosaka et al., 2023; Onu et al., 2023).

The consistent detection of SMX in this study, aligns with findings from a similar baseline study conducted in Kumasi   (Azanu et al., 2018). Azanu et al. (2018) demonstrated the presence of 12 antibiotics including amoxicillin, tetracycline and SMX in influents and effluents of WSPs, hospital wastewater and irrigated vegetables in Kumasi. They noted that SMX was consistently present in all samples studied although at lower levels (0.103 –3.590 μg/L) in comparison to the observation in this study. In this study, we only had resources to track SMX, which other studies suggest is the most prevalent pharmaceutical contaminants in Sub‐Saharan African environments (Munk et al., 2022; Segura et al., 2015). It was noted that KATH, which receives untreated hospital wastewater, contained higher concentrations of SMX compared to the other samples, with a quantified value of 0.127 mg/L. Ngigi et al. (2020) also found similar results, noting that the concentration of SMX in hospital wastewater was three times higher than the levels in samples from wastewater treatment plant and surface water. KATH is also where we recovered two multi‐drug resistant ST147 *K. pneumoniae* isolates. Our single extensively‐resistant *P. aeruginosa* isolate was isolated from the same wastewater. Both resistant lineages carried a large number of resistance genes including *sul1, sul2* genes encoding for SMX, and typically found on mobile elements.

AMR in clinically relevant bacteria poses a major public health challenge. Strains of *K. pneumoniae* resistant to extended beta‐lactamases or carbapenems as well as carbapenem‐resistant *P. aeruginosa* are classified in the critical category for new antibiotics development and are prevalent in healthcare‐associated infections (Mulani et al., 2019; Navon‐Venezia et al., 2017; Tacconelli et al., 2018). *K. pneumoniae*, particularly hypervirulent lineages, have been associated with severe invasive infections such as lung infections, kidney abscesses and endophthalmitis (Catalán‐Nájera et al., 2017; Chew et al., 2017). According to the European AMR surveillance report by WHO Regional Office for Europe/European Centre for Disease Prevention and Control (2022), *K. pneumoniae* is increasingly resistant to Watch and Reserve antimicrobials, including third‐generation cephalosporins and carbapenems. Additionally, *P. aeruginosa*, known for its multi‐drug resistance, was identified as the second most common cause of bacterial co‐infection in coronavirus disease (COVID‐19) patients (Lansbury et al., 2020). With one‐third of *Enterobacteriaceae* infections attributed to *K. pneumoniae* isolates and the high mortality rates associated with multidrug‐resistant *P. aeruginosa* in nosocomial infections, the clinical importance of pathogens sequenced in this study must be underscored (Lansbury et al., 2020; Navon‐Venezia et al., 2017). The burden of AMR in *Klebsiella* spp. in Africa is particularly high in West Africa (Murray et al., 2022) and although this assessment is based on a few data, recent surveillance reports from Nigeria, Ghana and The Gambia corroborate this concern (Afolayan et al., 2021; Munk et al., 2022; Okomo et al., 2020; Osei Sekyere & Reta, 2020). In Ghana, carbapenemase‐producing *K. pneumoniae* are now been reported in clinical settings (Dwomoh et al., 2022).

We identified resistant clones of clinical importance belonging to *Klebsiella* and *Pseudomonas* species in this study and determining how such strains circulate through the environment is an emerging research priority. Genomic methods offer the granularity necessary to connect isolates from a variety of niches, even when the number of strains examined is small, as in this study (Ikhimiukor et al., 2022). Despite the possibilities that genomic methods offer, very few wastewater isolates have been studied in Ghana or elsewhere in West Africa. *K. pneumoniae* species is considerably broad and includes environmental, commensal and potentially pathogenic strains. Despite the small number of isolates identified in this study, we uncovered a broad spectrum of environmental bacterial species but with the *K. pneumoniae* isolates, only two multi‐locus sequence types, ST18 and ST147 representing distinct KL and O types were isolated from wastewater in four examined sites in this study. These capsular polysaccharides feature prominently amongst *Klebsiella* that cause human invasive disease (Gorrie et al., 2022; Ikhimiukor et al., 2023; Opstrup et al., 2023). Our data suggest that these sequence types are over‐represented in the wastewater even from this admittedly small sample. Additionally, we cultured from grab samples, instead of the more sensitive passive sampling methods using Moore's swabs (Bivins et al., 2022) and therefore the true prevalence, and diversity, of resistant lineages could be greater.

*K. pneumoniae* belonging to ST147 have been frequently isolated from clinical infections and health‐system wastewater (Rocha et al., 2022; Suzuki et al., 2020), companion animals (Baron et al., 2021; Ovejero et al., 2017) and from food production chains (Klaper et al., 2021). ST147 is widely regarded as a high‐risk multidrug‐resistant *K. pneumoniae* lineage (Damjanova et al., 2008; Peirano et al., 2020; Wyres et al., 2019). In tandem with our study, genomes belonging to this sequence type were phenotypically resistant to multiple antibiotics, and harboured genetic determinants conferring resistance to multiple drug classes, including the ESBL gene, *bla*_CTX‐M‐15_. Infection with ESBL‐producing *Klebsiella* spp. is widely documented to impact heavily on human morbidity and mortality (Maslikowska et al., 2016; Sianipar et al., 2019). The two ST147 genomes from KATH showed high genetic relatedness (having 48 SNPs difference in their core genome alignment) but possessed some differences in their AMR gene content (presence of *bla*_TEM_ and *bla*_TEM‐169_ in either genome) and plasmid replicon content (absence of Col4401 in GH‐AEI_C_15), thereby indicating that these were highly similar but not same strains.

Although having fewer resistance determinants compared to ST147, ST18 genomes possessed more virulence determinants than the ST147 strains. A search for publicly available *K. pneumoniae* ST18 genomes in BIGSdb (39,497 records) and Pathogenwatch (32,642 records) returned a total of 12 genomes (as at January 2024 without correcting for possible duplicates). These were genomes of isolates from humans (*n* = 6) and fish (*n* = 1) from Argentina (Knecht et al., 2022), Brazil, India, Malawi, Philippines (Carlos et al., 2021), The Netherlands and the United States. Due to the scarcity of publicly available information of this ST in Africa (except a single strain from Malawi), our study might be the first report of ST18 in West Africa, and from wastewater environment.

We observed physical linkage of antibiotic and heavy metal resistance genes on the same plasmids in ST147 (Baker‐Austin et al., 2006). This co‐resistance could account for the persistence of the organisms and maintenance of their genetic determinants in the environment (Baindara, 2019). MOB‐suite has been used in studies to infer plasmid content of *K. pneumoniae* strains from draft genome sequences (Dereeper et al., 2022; Kochan et al., 2022), however, we acknowledge that results presented are within limitations that MOB‐suite plasmid reconstruction tool is unable to work well on novel plasmids with poor sequence similarities to those contained in the database (Robertson & Nash, 2018). Hence, we do not conclude that the single plasmids detected represent the plasmids present in strains from this study. Long read sequence, which we were not able to perform, would provide better insights on plasmid and plasmid content.

Transmission of resistant organisms can occur in the environment (Huijbers et al., 2015). Amongst resistance reservoirs, effluents emanating from hospital and faecal‐impacted wastewater are important for evolution and dissemination of antimicrobial resistant bacteria and their genes (McCarthy et al., 2021). Studies have emphasized the clinical relevance of environmental *K. pneumoniae* (Rocha et al., 2022; Runcharoen et al., 2017). One example is the study by Rocha et al. (2022), which sought to determine the outcome of clinically relevant traits of third‐generation cephalosporin‐resistant *K. pneumoniae*, including ST147, from the clinic to wastewater. Despite the small size and therefore low representativeness of our data, our findings suggest that once antimicrobial resistant traits are acquired in *K. pneumoniae*, they may be preserved in the wastewater environment. This represents a significant public health risk as wastewater treatment may not be sufficient to eliminate multidrug resistant organisms (Fouz et al., 2020; Galvin et al., 2010; Hassen et al., 2020; Runcharoen et al., 2017), especially in developing countries where wastewater treatment is either non‐existent or only available at poor efficiencies (Metcalfe et al., 2017).

### Limitations of the study

We encountered significant limitations due to restricted funding, which narrowed our scope of analysis to very few isolates and only SMX, of potential antimicrobials that could have been sought. Additionally, we used grab samples, which are less sensitive for detecting microbial contaminants than Moore's swabs and other passive sampling methods (Bivins et al., 2022). For all of these reasons, the actual chemical and microbial contamination levels may be higher than we report.

## CONCLUSION

Recent modelling by Lewnard et al. (2024) determined that as many as 247,800 deaths attributable to AMR could be averted by improvements in water, sanitation and hygiene. In this study, we identified organic pollutants, the antibiotic sulfamethoxazole, and clinically significant antibiotic resistant bacteria, including AMR priority species; *K. pneumoniae* and *P. aeruginosa*, in wastewater samples from Kumasi, Ghana. Tested wastewater samples, excluding that from CWSP, exhibited high levels of BOD, COD, EC, TN and TSS, surpassing the Ghanaian EPA standards for safe discharge. Hospital wastewater (KATH) was particularly concerning, with the highest SMX concentration recorded (0.127 mg/L), and harbouring two of the four multidrug resistant *K. pneumoniae* isolates and the sole *P. aeruginosa* isolate sequenced in this study. These resistant bacteria carried plasmid‐borne mobile genes conferring resistance to antibiotics and heavy metals. Given these findings, the discharge of these wastewater samples poses serious environmental and public health risks. Appropriate treatment measures must be implemented prior to discharge to mitigate these hazards.

Based on the findings from this study, we recommend regular maintenance and upgrade to keep wastewater treatment facilities in optimal condition. Furthermore, advanced treatment processes like adsorption should be implemented in existing wastewater treatment plants which is effective for removing contaminants such as SMX. GEPA should enhance its surveillance framework to ensure comprehensive monitoring and effective management of emerging contaminants and could include SMX in its monitoring protocols due to its link to antibiotic resistance proliferation. We additionally recommend enhanced partnerships between public health authorities and environmental agencies which will foster holistic approaches to curb antibiotic resistance.

## AUTHOR CONTRIBUTIONS

**Amen Ekhosuehi:** Conceptualization; methodology; investigation; writing – original draft; formal analysis. **Odion O. Ikhimiukor:** Methodology; formal analysis; investigation; writing – original draft. **Helen Michelle Korkor Essandoh:** Conceptualization; supervision. **Nana Yaw Asiedu:** Conceptualization; supervision. **Isoken Tito Aighewi:** Supervision. **Gabriel Temitope Sunmonu:** Investigation; writing – review and editing. **Erkison Ewomazino Odih:** Methodology; investigation; writing – review and editing. **Anderson O. Oaikhena:** Methodology; investigation; supervision; writing – review and editing. **Dorothy Cyril‐Okoh:** Investigation; formal analysis. **Clara Yeboah:** Resources; methodology; conceptualization. **Iruka N. Okeke:** Writing – review and editing; supervision; resources; writing – original draft.

## CONFLICT OF INTEREST STATEMENT

The authors declare no conflicts of interest.

## Supporting information


**TABLE S1:** Analyte, physicochemical and microbial characteristics of wastewater samples.
**TABLE S2:** Minimum inhibitory concentration of *K. pneumoniae* strains from this study when tested against 19 antibiotics.
**TABLE S3:** Metadata and genetic determinants (AMR, plasmid replicons, virulence) of genomes from this study.
**TABLE S4:** Metadata and genetic determinants (AMR, plasmid replicons, virulence) of genomes from Pathogenwatch.
**TABLE S5:** Single nucleotide polymorphisms distances of *K. pneumoniae* ST18 and ST147 genome alignments.
**TABLE S6:** Information, AMR genes and heavy metal resistance genes of reconstructed plasmids from draft genomes in this study.

## Data Availability

All raw genome sequences of the isolates characterized in this study have been submitted to the European Nucleotide Archive (https://www.ebi.ac.uk/ena/browser/) under the study accession number PRJEB58695. The K. pneumoniae genome sequences are available under the following sample accession numbers: ERS14392544, ERS14392545, ERS14392546, ERS14392547, ERS14392548, ERS14392549, ERS14392550.
